# Sequential Irreversible Electroporation for Locally Advanced Pancreatic Cancer

**DOI:** 10.3390/diagnostics13223458

**Published:** 2023-11-16

**Authors:** Daniel Yuxuan Ong, Uei Pua

**Affiliations:** Department of Diagnostic Radiology, Tan Tock Seng Hospital, Singapore 308433, Singapore; dadadachow@gmail.com

**Keywords:** irreversible electroporation (IRE), locally advanced pancreatic cancer (LAPC)

## Abstract

Pancreatic cancer is a lethal disease, with locally advanced pancreatic cancer (LAPC) having a dismal prognosis. For patients with LAPC, gemcitabine-based regimens, with or without radiation, have long been the standard of care. Irreversible electroporation (IRE), a non-thermal ablative technique, may potentially prolong the survival of patients with LAPC. In this article, the authors present a case of LAPC of the uncinate process (biopsy proven pancreatic neuroendocrine carcinoma) with duodenal invasion. The patient had a combination of chemotherapy and radiation therapy but was found to have stable disease. He then underwent intra-operative IRE with cholecystectomy, Roux-en-Y gastrojejunostomy and hepaticojejunostomy. He subsequently underwent percutaneous IRE 13 months post open IRE. The patient also completed peptide receptor radionuclide therapy and has been started on Lanreotide. Following combination therapy, the pancreatic tumor showed significant reduction in size, with patient survival at 53 months post-diagnosis at the time of writing.

Pancreatic cancer is a highly lethal disease, with locally advanced pancreatic cancer (LAPC) having a dismal prognosis. Approximately 30% of patients present with LAPC, defined as greater than 180° circumference tumor encasement of the superior mesenteric or celiac artery, or non-reconstructable venous involvement [1].

For patients with LAPC, gemcitabine-based regimens, with or without radiation, have long been the standard of care. The use of 5-fluorouracil, leucovorin, irinotecan, and oxaliplatin (FOLFIRINOX) chemotherapy has improved survival, but the overall prognosis remains poor. Irreversible electroporation (IRE), a non-thermal ablative technique, may potentially prolong the survival of patients with LAPC [2,3].

A 59-year-old man with an Eastern Cooperative Oncology Group (ECOG) performance status of 0 presented with LAPC of the uncinate process (biopsy proven pancreatic neuroendocrine carcinoma) with duodenal invasion (Figure 1A,B). The patient underwent a combination of chemotherapy and radiation therapy but was found to have stable disease. 

The initial plan was for debulking pancreaticoduodenectomy (Whipple’s procedure) with marginal accentuation IRE. Intra-operatively, it was found that there was extensive local invasion. The procedure was thus converted to intra-operative IRE (Figure 2) with cholecystectomy, Roux-en-Y gastrojejunostomy and hepaticojejunostomy. Following open IRE, perforation of the duodenum was encountered (Figure 3), but the patient remained asymptomatic, likely due to gastrojejunostomy. The pancreatic mass 1 year post open IRE showed reduction in size (Figure 4A,B). The patient subsequently underwent percutaneous IRE 13 months post open IRE (Figure 5). The patient also completed peptide receptor radionuclide therapy and has been started on Lanreotide. 

Following the combination therapy, the pancreatic tumor showed significant reduction in size (Figure 6A,B), with patient survival at 53 months post-diagnosis at the time of writing. 

Given the poor survival of patients with LAPC, even without early distant metastases, several groups are focusing on combining systemic chemotherapy with local ablative therapies. IRE is a non-thermal ablative technique in which high-voltage electrical pulses are applied between needle electrodes. The pulses irreversibly damage the cellular membrane by creating nanopores, inducing programmed cell death [4]. IRE has been shown to be promising in terms of overall survival for patients with LAPC [3]. In addition, percutaneous IRE seems to prolong survival compared with standard of care, as demonstrated in the PANFIRE-2 study [2]. 

This case demonstrates that sequential IRE may play a role in sustained local tumor response and control for LAPC (pancreatic neuroendocrine carcinoma). 

## Figures and Tables

**Figure 1:**
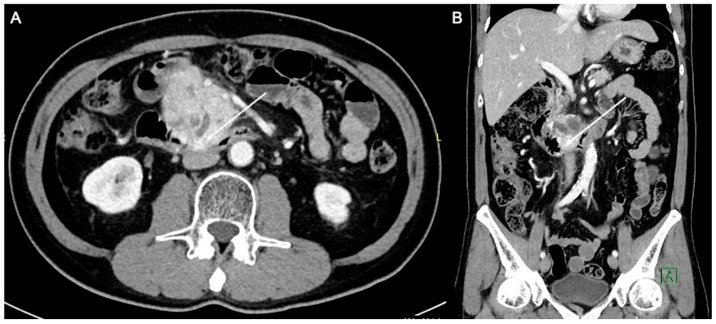
Axial (**A**) and coronal (**B**) computed tomography images of the patient demonstrating a heterogeneously enhancing pancreatic mass measuring approximately 6.3 × 4.4 cm. There is duodenal involvement by the pancreatic mass (arrows).

**Figure 2:**
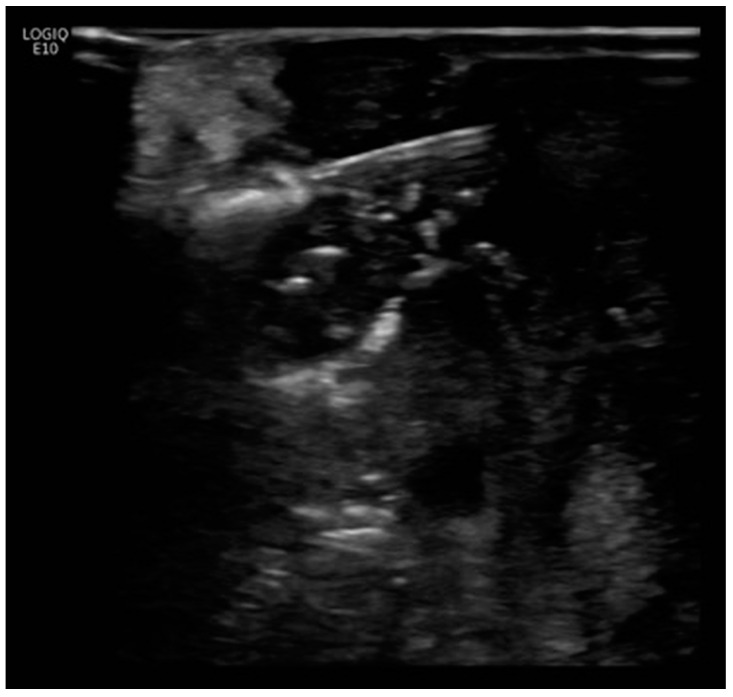
Intra-operative ultrasound image. Three parallel probe arrays were utilized for open irreversible electroporation (IRE). Open IRE procedural details: Three parallel probe arrays were positioned from medial to lateral, starting from the region besides the superior mesenteric artery. A total of six ablation positions, each with 1 cm tip exposure and five pull backs at each location, was performed. The region besides the duodenal c-loop was avoided to prevent bowel perforation. Ablation using between 80 and 160 pulses at 2000–2500 Volts was delivered at each location (with 12 A rise in current) and monitored under intra-operative ultrasound.

**Figure 3:**
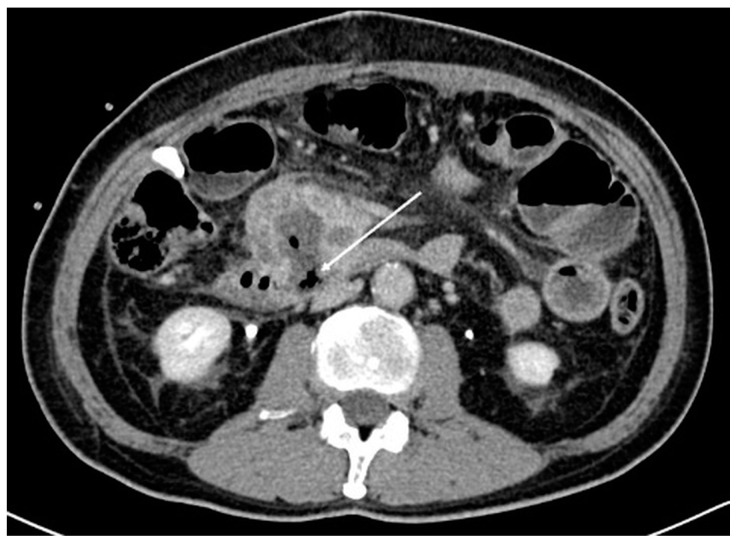
Axial computed tomography image post open irreversible electroporation. At the location of the tumor invasion of the duodenum, there is a defect in the wall with gas seen (arrow). This likely represents perforation of the duodenum due to breakdown of the ablated tumor.

**Figure 4:**
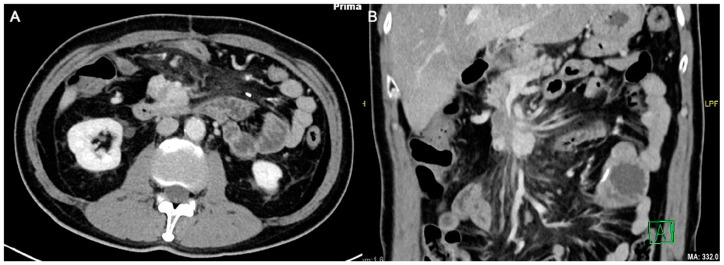
Axial (**A**) and coronal (**B**) computed tomography images 1 year post open irreversible electroporation. The pancreatic mass now measures approximately 3.9 × 2.6 cm.

**Figure 5:**
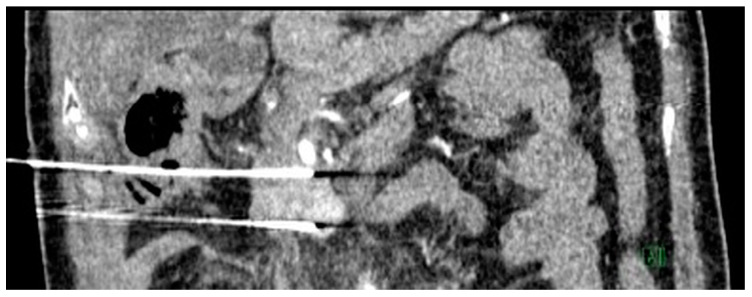
Intra-operative coronal computed tomography (CT) image demonstrating two irreversible electroporation (IRE) probes inserted in the cranio-caudal orientation into the tumor bed. Percutaneous IRE procedural details: Two IRE probes were inserted in the cranio-caudal orientation into the tumor bed under CT guidance, ensuring the needle is 2 cm apart and parallel—unsheathed 1.5 cm. Two cycles of 90 pulses at 2500 V performed with 12 A rise in current (initial 10 test pulses performed). This was followed by withdrawal of the needles proximally by 1.5 cm with another two cycles of 90 pulses. Post-ablation scan showed no active bleed and air locules seen around the surgical bed secondary to hydrolysis. Needles were then removed.

**Figure 6:**
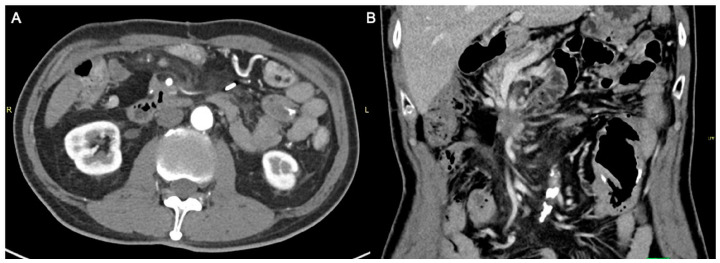
Axial (**A**) and coronal (**B**) computed tomography images demonstrating the remnant pancreatic tumor, now smaller in size and extent, measuring approximately 2.3 cm.

## Data Availability

Not applicable.
